# Psychometric evaluation of the Korean version of the Diabetes Symptom Checklist-Revised (DSC-R) for Patients with Type 2 Diabetes

**DOI:** 10.1186/1477-7525-12-77

**Published:** 2014-05-19

**Authors:** Eun-Hyun Lee, Kwan-Woo Lee, Rhayun Song, Frank J Snoek, Seung Hei Moon

**Affiliations:** 1 Graduate School of Public Health, Ajou University, 164 Worldcup-ro, Yeongtong-gu, Suwon-si, Gyeonggi-do 443-380, Republic of Korea; 2 Department of Endocrinology and Metabolism, School of Medicine, Ajou University, Suwon, Republic of Korea; 3 College of Nursing, Chungnam National University, Daejeon, Republic of Korea; 4 Department of Medical Psychology and Medical Social Work, VU University Medical Center, Amsterdam, Netherlands; 5 Graduate School, Inha University, Incheon, Republic of Korea

**Keywords:** Diabetes, Symptom, Reliability, Validity, Korea

## Abstract

**Background:**

This study was to elucidate the psychometric properties of the Korean version of the Diabetes Symptom Checklist-Revised (K-DSC-R), which is a patient-reported outcome measure of diabetes symptom burden.

**Methods:**

A sample of 432 Korean patients with diabetes was recruited from university hospitals. The data were analyzed using exploratory factor analysis (EFA), confirmatory factor analysis (CFA), multitrait/multi-item correlation, Pearson’s correlation, *t*-test, ANOVA, and Cronbach’s alpha for construct, item-convergent/discriminant, concurrent, and known-groups validity, and internal consistency reliability.

**Results:**

EFA extracted a total of 29 items clustered into 7 subscales from the K-DSC-R. The construct of the seven-subscales was supported by CFA. The scaling success rates of item-convergent validity were 100% for all subscales, and those of item-discriminant validity ranged from 83.3% to 100%. Patients in more-depressed groups and in the HbA1c-uncontrolled group had higher K-DSC-R scores, satisfying the known-groups validity. The subscales of the K-DSC-R were moderately correlated with health-related quality of life, indicative of the established concurrent validity. The Cronbach’s alpha of the K-DSC-R was 0.92.

**Conclusions:**

The psychometric properties of the K-DSC-R have been established. It is thus appropriate for use with respect to reliability and validity in practice and clinical trials for Korean patients with type 2 diabetes.

## Background

Diabetes has reached epidemic levels. In 2012 about 371 million people in the world had diabetes, and this number is expected to rise to 522 million by 2030, increasing in every country [1]. According to the Korean National Health and Nutrition Examination Survey [2], the prevalence of diabetes among adults aged ≥ 30 years was 9.9% in 2012. The reported prevalence was increased with age, being 1.9%, 5.0%, 12.6%, 20.3%, and 22.0% among individuals in their 30s, 40s, 50s, 60s, 70s and over, respectively.

Patients with type 2 diabetes experience common symptoms (e.g., excessive thirst, dryness of mouth, fatigue, difficulty in thinking, and drowsiness) along with the fluctuation of their blood glucose levels. They may also experience other symptoms such as visual blurring, numbness and tingling in the extremities, and calf pain due to long-term diabetic complications [3], and may suffer multiplex symptoms throughout the trajectory of the disease process.

Symptoms are perceived subjectively in terms of what the individual believes they mean in the physical and psychosocial-spiritual contexts [4]. The attribute of “subjective perception” implies that a symptom is better assessed directly by the patient than, for example, by a clinician. The assessment of patient-reported outcome (PRO) has recently been emphasized in determining the efficacy of new therapies. In particular, PRO measurements are becoming more important in the field of treatment effects. For example, the United States Food and Drug Administration (2009) incorporated PRO instruments [e.g., measuring symptom or health-related quality of life (HRQOL)] in the decision-making process regarding the effect of a medical intervention in clinical trials [5].

The Diabetes Symptom Checklist–Revised (DSC-R) is a self-reported questionnaire measuring the occurrence and perceived discomfort of symptoms associated with type 2 diabetes and its potential complications. The DSC-R comprises 34 items clustered into 8 subscales: psychological fatigue, psychological cognitive, neuropathic pain, sensory neuropathic, cardiovascular, ophthalmologic, hyperglycemic, and hypoglycemic subscales. The scale measures the comprehensive symptom burden of type 2 diabetes. The psychometric properties of the DSC-R are empirically well established in patients with type 2 diabetes [6].

The DSC-R has been translated into many languages, and there are currently 47 versions (http://www.mapi-trust.org). Nevertheless, the psychometric properties of the translated versions have rarely been reported. From a measurement perspective, it is recommended that the psychometric properties of an instrument that has been translated for use in new cultures or languages should be re-evaluated in a population that is representative of the target culture or language [7]. Conceptually, the perception of a symptom can be affected by an individual’s belief about illness or ethnicity in the culture in which the individual resides [8]. Therefore, the psychometric properties of a new translated version of the DSC-R need to be verified before it is used in either practice or research involving a target language or culture.

The Korean version of the DSC-R (K-DSC-R) has already been produced, but its reliability and validity have never been reported in Korean patients with type 2 diabetes. The purpose of the present study was thus to elucidate the reliability and validity of the K-DSC-R in patients with type 2 diabetes.

## Methods

### Study design

A cross-sectional and methodological research design was used to evaluate the following psychometric properties of the K-DSC-R: internal consistency reliability, factorial construct validity, item-convergent and discriminant validity, concurrent validity, and known-groups validity.

### Sample and data-collection procedure

A convenience sample of 432 subjects was recruited from 2 university hospitals in South Korea, between July and November, 2012. The number of participants met the preferred sample size of 5–10 times per item for factor analysis [9]. Patients who were diagnosed with type 2 diabetes by physicians, aged at least 20 years, and articulate in the Korean language were invited to participate in the study after receiving approval for the protocol from the institutional review boards of the two university hospitals (Ajou University Hospital and Chungnam National University, College of Nursing) at which the participants were enrolled.

Potential subjects were recruited from outpatient clinics at two university hospitals by health professionals and briefly informed about the study. If they agreed to participate, they then met with the research assistants who informed them about the purpose of this study and the nature of their participation. Potential subjects who were articulate and agreed to participate were asked to sign a formal consent form and to complete the self-reported questionnaires.

### Measures

#### The K-DSC-R

The DSC-R, which originated from the DSC-Type 2 that was validated in patients with type 2 diabetes from the Netherlands [10], comprises 34 items that are clustered into 8 subscales. Each item asks whether or not the respondent has a particular symptom in the past month. If the respondent answers “yes”, they are asked to rate how troublesome that particular symptom is to him/her on a 5-point Likert scale from 1 (not at all) to 5 (extremely). The item scores are transformed from 1–5 to 0–4, with a score of 0 also applying where a symptom does not occur. The total and all subscale scores are summed and divided by the total number of items in the total and subscales. Higher scores indicate a greater symptom burden. The psychometric properties of the DSC-R have been established [6]. The Mapi Research Trust (http://www.mapi-trust.org) translated the DSC-R into Korean using a translation and back-translation technique. The K-DSC-R used in this study was obtained from the Mapi Research Trust after obtaining the permission of the original author of the DSC-R questionnaire.

#### The Diabetes-Specific Quality of Life (D-QOL) questionnaire

The D-QOL is a self-reported questionnaire that comprises 16 items requiring a response on a 5-point Likert scale, where a higher score indicates a better HRQOL. Its content validity, factorial validity, concurrent validity, known-groups validity, and internal consistency reliability have been established in 402 Korean patients with diabetes [11].

#### The Center for Epidemiologic Studies-Depression (CES-D) scale

The CES-D scale is a widely-used and well-validated measure of depression, originally developed for the general population [12]. It comprises 20 items scored on a 4-point scale, where scores of 0–9, 10–15, 16–24, and >24 imply no depression, mild depression, moderate depression, and severe depression, respectively. Internal consistency reliability, test-retest reliability, concurrent validity, and discriminant validity of this tool have been established in a Korean population [13].

#### HbA1c control status

Glycemic control was determined by HbA1c categorized into two groups, ‘controlled’ (HbA1c <7.0% [53 mmol/mol]) and ‘uncontrolled’ (HbA1c ≥7.0% [53 mmol/mol]), based on regular laboratory medical data obtained by high-performance liquid chromatography [14].

### Data analysis

PASW statistics software (version 18) was used to analyze the data. Descriptive statistics were computed for the item completeness of the K-DSC-R. The factorial validity of the K-DSC-R scale was tested using both exploratory factor analysis (EFA) and confirmatory factor analysis (CFA). EFA was performed to empirically identify the structure underlying the K-DSC-R with Korean patients with type 2 diabetes. Before conducting EFA, Bartlett’s test of sphericity and the Kaiser-Mayer-Olkin (KMO) measure of sampling adequacy were performed to justify undertaking EFA [15]. EFA was conducted using principal-components analysis with varimax rotation. After the rotation, factors with an eigenvalue greater than 1 were retained. The meaningful loading criterion for the factors was set at ≥0.50 [16]. Correlation coefficients among extracted factors were computed using Pearson’s correlation to identify the potentiality of collapsing factors due to a strong correlation [17].

CFA was conducted to confirm whether or not the underlying structure derived from EFA was supported. The model parameters for CFA were estimated using the maximum-likelihood method. The goodness-of-fit of the model was evaluated based on the chi-square test and the following multiple indices: ratio of chi-square value to the degrees of freedom (CMIN/DF), root-mean-square residual (RMR), goodness-of-fit index (GFI), root-mean-square error of approximation (RMSEA), comparative-fit index (CFI), and incremental-fit index (IFI). Values of <3.0, <0.50, and <0.60 for the CMIN/DF ratio, RMR, and RMSEA, respectively, indicate a good fit. A value of >0.90 for GFI, CFI, and IFI served as a cutoff for an acceptable fit [18,19].

Item-convergent and discriminant validity of the K-DSC-R was tested using the multitrait/multi-item correlation matrix [20]. For the correlation matrix, Pearson’s correlation coefficients of an item with its own scale (corrected for overlap) and other scales were computed. Item-convergent validity is satisfied if the correlation coefficient for an item and its own scale is ≥0.40, while item-discriminant validity is satisfied if the correlation coefficient between an item and its own scale is higher (by more than two standard errors) than the correlation coefficients between that item and the other scales.

For the test of known-groups validity, it was hypothesized – based on previous studies [21,22] – that the symptom distress as measured using the K-DSC-R would be higher for patients in the more-depressed groups. In addition, it was hypothesized that there would be greater symptom distress among patients in the HbA1c-uncontrolled group [6]. These tests were analyzed using *t*-tests or ANOVA. The effect size of known-groups validity was assessed using Cohen’s *d* and squared eta value (*η*^2^).

For convergent validity it was hypothesized that K-DSC-R scores would be at least moderately correlated with the HRQOL score as indicated by *r* = 0.64-0.53 on the previous study [23]. This hypothesis was tested using Pearson’s correlation coefficient.

Internal consistency reliability was evaluated by computing Cronbach’s alpha coefficient, with the acceptability criterion set at ≥0.70 [24].

## Results

### Characteristics of the subjects

The subjects who participated in this study comprised 216 males and 216 females, aged 61.21 ± 10.66 years (mean ± SD). Most of the patients were married (83.1%, *n* = 359), approximately two-thirds (60.7%, *n* = 262) had graduated from high school or above, and 40.7% (*n* = 176) were employed. The duration of type 2 diabetes was 12.77 ± 8.27 years. The proportions of subjects receiving an oral hypoglycemic agent (OHA) alone, insulin injection alone, OHA in combination with insulin injection, and diet/exercise without medication for the treatment for their diabetes were 62.7% (*n* = 271), 10.0% (*n* = 43), 25.0% (*n* = 108), and 2.3% (*n* = 10), respectively.

### Completeness of data

The rate of missing values for each item of the K-DSC-R was very low (0.0–0.2%). The missing values were imputed with the mean of all non-missing values of its subscale, in accordance with the scoring guidelines of the DSC-R.

### Factorial construct validity

Bartlett’s test of sphericity was significant (χ^2^ = 6674.75, *p* < 0.001), and the KMO index was 0.91, which indicates that the data were suitable for EFA. Initial principal-components analysis with varimax rotation extracted an eight-factor solution (eigenvalue >1), which explained 62.4% of the total variance. However, five items (items 2, 5, 18, 29, and 31) did not meaningfully load onto any factors at a criterion cutoff of ≥0.50. These five items were eliminated before conducting a subsequent principal-components analysis, which extracted a seven-factor solution that accounted for 62.68% of the total variance (Table 1). Pearson’s correlation coefficients among the seven factors ranged from low (*r* = 0.275) to moderate (*r* = 0.636), implying that the subscales were related but not redundant thus, the factors did not need to be collapsed. Each factor was labeled from F1 to F7 (based on the names of the subscales in the DSC-R) as neuropathic pain, psychological fatigue, hypoglycemic, ophthalmologic, hyperglycemic, cardiovascular, and sensory neuropathic subscales, respectively.

**Table 1:** Exploratory factor analysis and internal consistency reliability of the K-DSC-R

		**Factors and item loadings**						
	**Item**^**a**^	**F1**	**F2**	**F3**	**F4**	**F5**	**F6**	**F7**
15	Burning pain: calves	**0.761**	0.108	0.103	0.131	0.004	−0.124	0.176
21	Shooting pain: legs	**0.747**	0.200	0.068	0.095	0.180	0.096	0.068
25	Burning pain: legs	**0.726**	0.256	0.092	0.115	0.054	−0.038	0.139
34	Tingle/prickle: legs or feet	**0.683**	−0.112	0.077	0.016	0.003	0.151	0.203
11	Tingle: limbs	**0.637**	0.240	0.054	0.074	0.106	0.234	−0.023
26	Tingle/prickle: hands or fingers	**0.581**	0.046	0.104	0.007	0.085	0.280	0.077
4	Overall fatigue	0.202	**0.754**	0.205	0.109	0.185	0.081	0.148
1	Energy	0.130	**0.725**	0.154	0.142	0.112	0.077	0.202
20	Fatigue: when getting up	0.157	**0.620**	0.339	0.121	0.135	0.102	−0.011
6	Sleepiness	0.049	**0.601**	0.186	0.236	0.138	0.072	−0.073
17	Fatigue: daytime	0.190	**0.536**	0.222	0.177	0.273	0.206	0.086
27	Irritation	0.112	0.118	**0.692**	0.143	0.118	0.321	0.027
19	Irritability: before a meal	0.080	0.202	**0.688**	0.053	0.109	0.072	−0.011
7	Concentration	0.128	0.384	**0.667**	0.283	0.141	−0.073	0.148
8	Mood	0.102	0.213	**0.606**	0.208	0.094	0.295	0.081
33	Attention	0.114	0.199	**0.599**	0.232	0.105	0.048	0.257
14	Vision: getting worse	0.042	0.155	0.196	**0.773**	0.129	0.097	−0.034
10	Vision: blurred	0.113	0.224	0.057	**0.759**	0.111	0.229	0.193
28	Vision: sudden deterioration	0.111	0.159	0.292	**0.730**	0.039	−0.113	0.084
22	Vision: alternating clear and blurred	0.126	0.131	0.134	**0.719**	0.136	0.232	0.144
32	Drinking	0.056	0.121	0.058	0.073	**0.801**	0.046	−0.083
12	Thirsty	0.150	0.165	0.238	0.063	**0.752**	0.125	0.194
16	Dry mouth	0.031	0.191	0.138	0.044	**0.729**	0.201	0.249
23	Urination	0.105	0.138	0.054	0.182	**0.689**	−0.029	−0.044
13	Palpitations: heart	0.100	0.192	0.141	0.082	0.065	**0.769**	0.091
24	Pain: chest	0.184	0.012	0.212	0.161	0.195	**0.675**	0.020
30	Breath: shortness	0.232	0.401	0.096	0.178	0.191	**0.508**	0.240
3	Numbness: feet	0.284	0.110	0.106	0.121	0.106	0.045	**0.796**
9	Numbness: hands	0.259	0.117	0.138	0.155	0.050	0.154	**0.718**
Cronbach’s alpha		0.81	0.82	0.81	0.83	0.79	0.66	0.71

^a^Only the main expression items are presented.

Values in boldface are meaningful factor loadings (≥0.50).

F1, neuropathic pain factor; F2, psychological fatigue factor; F3, hypoglycemic factor; F4, ophthalmologic factor; F5, hyperglycemic factor; F6, cardiovascular factor; F7, sensory neuropathic factor.

**Figure 1:**
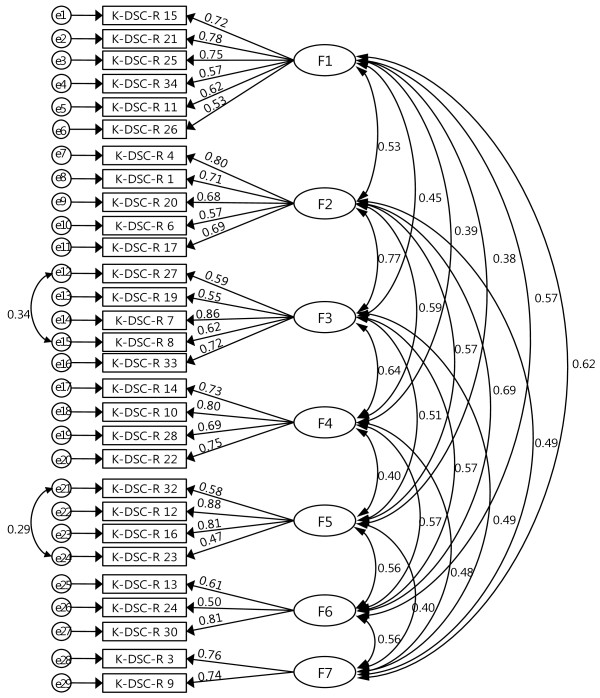
F1, neuropathic pain factor; F2, psychological fatigue factor; F3, hypoglycemic factor; F4, ophthalmologic factor; F5, hyperglycemic factor; F6, cardiovascular factor; F7, sensory neuropathic factor.

CFA was performed with the underlying seven-factor model. The initial model fit indices were as follows: χ^2^(356) = 943.080, CMIN/DF = 2.649, RMR = 0.035, GFI = 0.868, RMSEA = 0.062, CFI = 0.888, and IFI = 0.889. These indices implied partial failure of the acceptable criteria for the model fit, since the values were close to their cutoff criteria; modification indices (MIs) were therefore computed. Based on the largest covariance suggested by the MIs, two error terms were subsequently connected with two-headed curved arrows (Figure 1). As a result, χ^2^(354) was meaningfully decreased to 867.354. The modified model fit indices were improved as follows: CMIN/DF = 2.450, RMR = 0.035, GFI = 0.877, RMSEA = 0.058, CFI = 0.902, and IFI = 0.907, indicating that the revised seven-factor model fitted the data well overall. Figure 1 shows the standardized loadings of this model. All values except item 23 (which was below the criterion of 0.5) significantly loaded onto the seven latent factors, ranging from 0.51 to 0.883. Item 23 (“Frequent need to empty your bladder?”) is a representative condition for diabetes [3] and was therefore retained.

### Item-convergent and item-discriminant validity

As presented in Table 2, item-factor correlations between items and their own factors (after correcting for overlap) ranged from 0.501 to 0.732. Thus, all items satisfied item-convergent validity (cutoff of 0.40), and the scaling success rates of the item-convergent validity were all 100%. There were several failed cases for item-discriminant validity, so that the scaling success rates of item discrimination were 93.3% for factors 2 and 3, 83.3% for factor 6, and 100% for factors 1, 4, 5, and 7.

**Table 2:** Item-convergent and item-discriminant validity

	**Subscale**						
**Item no.**	**F1**	**F2**	**F3**	**F4**	**F5**	**F6**	**F7**
15	**0.621**	0.282	0.262	0.248	0.149	0.245	0.396
21	**0.652**	0.381	0.314	0.266	0.317	0.424	0.374
25	**0.623**	0.402	0.317	0.269	0.223	0.312	0.385
34	**0.545**	0.167	0.179	0.142	0.114	0.254	0.351
11	**0.558**	0.393	0.295	0.250	0.254	0.363	0.305
26	**0.503**	0.279	0.250	0.187	0.201	0.330	0.331
4	0.394	**0.732**	0.515	0.382	0.410	0.423	0.349
1	0.333	**0.635**	0.463	0.388	0.345	0.394	0.319
20	0.313	**0.597**	*0.523*	0.372	0.349	0.387	0.245
6	0.208	**0.495**	*0.430*	0.373	0.309	0.306	0.174
17	0.362	**0.593**	0.492	0.409	0.436	0.468	0.298
27	0.281	0.438	**0.614**	0.378	0.306	0.440	0.268
19	0.209	*0.414*	**0.503**	0.303	0.258	0.304	0.214
7	0.305	*0.609*	**0.687**	0.496	0.366	0.347	0.324
8	0.278	0.473	**0.617**	0.422	0.308	0.458	0.285
33	0.289	0.467	**0.580**	0.415	0.290	0.350	0.340
14	0.185	0.394	0.426	**0.666**	0.278	0.322	0.211
10	0.304	0.445	0.435	**0.689**	0.307	0.418	0.382
28	0.226	0.400	0.447	**0.625**	0.213	0.243	0.265
22	0.287	0.402	0.445	**0.645**	0.302	0.405	0.307
32	0.158	0.326	0.235	0.210	**0.602**	0.232	0.112
12	0.318	0.438	0.435	0.311	**0.680**	0.407	0.305
16	0.239	0.429	0.356	0.281	**0.633**	0.388	0.288
23	0.184	0.336	0.260	0.260	**0.501**	0.193	0.163
13	0.284	0.338	0.381	0.279	0.247	**0.567**	0.249
24	0.309	0.290	*0.364*	0.295	0.235	**0.443**	0.227
30	0.420	*0.532*	*0.445*	0.421	0.382	**0.517**	0.398
3	0.432	0.317	0.332	0.296	0.254	0.328	**0.556**
9	0.425	0.335	0.342	0.332	0.230	0.362	**0.556**

Boldface values indicate meaningful item-convergent validity.

Italic values indicate failed item-discriminant validity.

### Known-groups validity

The known-groups validity data are presented in Table 3. The K-DSC-R scores differed significantly among the depressed groups (*F* = 78.89, *p* < 0.001, *η*^2^ = 0.35). As hypothesized, patients in the more-depressed groups had higher K-DSC-R scores. Known-groups validity was also tested by evaluating the HbA1c control status using the *t*-test. The mean K-DSC-R score was significantly lower in the HbA1c-controlled group (*t* = −2.13, *p* = 0.03, *d* = 0.65). These findings support the known-groups validity of the K-DSC-R.

**Table 3:** Known-groups validity: mean differences in K-DSC-R Scores according to depression classification and HbA1c control status

	***n***	**Mean (SD)**	***F *** **or *t *** **(*p*)**	**Post-hoc test**
Depression classification				
None^a^	189	1.50 (1.85)	36.79^e^ (*p* < 0.001)	a, b < c < d (Dunnett’s T3)^f^
Mild^b^	124	2.29 (2.29)		
Moderate^c^	79	4.00 (2.96)		
Severe^d^	37	8.31 (4.96)		
HbA1c status				
Controlled (HbA1c <7.0%)	145	2.32 (2.74)	−2.13 (*p* = 0.03)	
Uncontrolled (HbA1c ≥7.0%)	287	3.03 (3.46)		

^a^No depression.

^b^Mild depression.

^c^Moderate depression.

^d^Severe depression.

^e^Welch’s test was computed due to the heterogeneous variance for ANOVA.

^f^Dunnett’s T3 comparison was conducted due to the nonequal variances for post-hoc testing.

### Concurrent validity

The K-DSC-R total score was significantly and negatively correlated with the overall D-QOL score (*r* = −0.72, *p* < 0.001). The correlation coefficients between the subscales of the K-DSC-R and D-QOL ranged from −0.41 to −0.62 (*p* < 0.001 for all). These results satisfied the hypothesized moderate correlation between K-DSC-R and HRQOL, supporting the concurrent validity of the K-DSC-R.

### Internal consistency of reliability

Cronbach’s alpha coefficient of the K-DSC-R was very high, at 0.92; the coefficients of all of the subscales except factor 6 (Cronbach’s alpha = 0.66) were above the criterion of 0.70 (Table 1).

## Discussion

The present study evaluated the psychometric properties of the K-DSC-R – which was designed to measure the occurrence and perceived discomfort of symptoms associated with diabetes and its potential complications – in Korean patients with type 2 diabetes. This culturally adapted instrument exhibited good reliability and validity.

Different from the original 34-item DSC-R covering 8 domains, in this study the K-DSC-R comprised 7 subscales with a total of 29 items: 6 for the neuropathic pain subscale, 5 for the psychological fatigue subscale, 5 for the hypoglycemic subscale, 4 for the ophthalmologic subscale, 4 for the hyperglycemic subscale, 3 for the cardiovascular subscale, and 2 items for the sensory neuropathic subscale. This seven-subscale construct was well validated using both EFA and CFA. However, there was some discrepancy in the underlying construct between the DSC-R and K-DSC-R (Table 4). The psychological cognitive subscale (items 6, 7, 31, and 33) in the DSC-R was not maintained as an independent subscale in the K-DSC-R. In this study item 31 (“Fuzzy feeling in your head?”) was eliminated from the statistical factor analysis, and item 6 (“Sleepiness and drowsiness?”) was loaded onto the psychological fatigue subscale of the K-DSC-R. This was congruent with a previous study finding involving USA diabetes patients that item 6 was loaded onto the psychological fatigue subscale rather than the psychological cognitive subscale [25]. The clustering of item 6 with the psychological fatigue subscale was not unexpected, since the psychological fatigue and cognitive subscales were subdivided in the psychological dimension in the original DSC-Type 2 [10]. Unlike item 6, items 7 (“Difficulty concentrating?”) and 33 (“Difficulty paying attention?”) were clustered together with the hypoglycemic subscale in the present study. Even though different patients can experience a range of different symptoms when their blood glucose declines, these two items were noted as being common symptoms of hypoglycemia (e.g., difficulty in thinking, anger, sadness, hunger, headache, and dizziness) [3]. It was thus reasonable to group these items within the hypoglycemic subscale in the present study. Regarding the discrepancy of psychological cognitive subscale, a cultural difference also needs to be considered a potentiality, since there may exist cultural differences in cognition between Eastern Asians (Korea, Japan, and Taiwan) and Western people (USA) [26].

**Table 4:** Comparison of subscales and their items for both the original DSC-R and the K-DSC-R

**Item no.**	**DSC-R subscale**								**K-DSC-R subscale**						
	**PF**	**PC**	**NP**	**NS**	**C**	**O**	**HO**	**HE**	**PF**	**NP**	**NS**	**C**	**O**	**HO**	**HE**
1	●								●						
4	●								●						
17	●								●						
20	●								●						
6		●							○						
7		●												○	
31		●													
33		●												○	
2			●												
15			●							●					
21			●							●					
25			●							●					
3				●							●				
9				●							●				
11				●						○					
26				●						○					
29				●											
34				●						○					
5					●										
13					●							●			
24					●							●			
30					●							●			
10						●							●		
14						●							●		
18						●									
22						●							●		
28						●							●		
8							●							●	
19							●							●	
27							●							●	
12								●							●
16								●							●
23								●							●
32								●							●

● Symbol showing subscales and their own items: items included in the same subscales in both the DSC-R and K-DSC-R.

○ Symbol showing subscales and their own items: items placed in different subscales in the K-DSC-R and the DSC-R.

PF, psychological fatigue factor; PC, psychological cognitive factor; NP, neuropathic pain factor; NS, sensory neuropathic factor; C, cardiovascular factor; O, ophthalmologic factor; HO, hypoglycemic factor; HE, hyperglycemic factor.

The sensory neuropathic subscale of the DSC-R consists of six items (3, 9, 11, 26, 29, and 34). In the present study, items 3 and 9 were retained in the sensory neuropathic subscale (Table 4). However, items 11, 26, and 34 were placed on the neuropathic pain subscale of the K-DSC-R, which is perhaps not surprising from a linguistic perspective since these three items involve “tingling” or “prickling” in the limbs, hands, or feet. The Korean language contains approximately 90 vocabularies for expressing the types and intensity of pain. “Tingle” and “prickle” (which translate to “soo shi da” and “wook shin gau ri da,” respectively, in Korean) are terms that Koreans typically use to express “pain” [27]. It is therefore understandable that the three items should be clustered with the neuropathic pain subscale rather than items about numbness (loss of sensation) in the feet or hands of the neurological sensory subscale. This discrepancy may thus have arisen as a result of language differences.

In the item-level tests for the item-convergent/discriminant validity, the item-convergent validity of the K-ADS-R was 100% successful, with item-subscale correlation coefficients ranging from 0.443 to 0.732. This is similar to the range of 0.44–0.78 quoted in a DSC-R validation study [6]. However, the item-discriminant validity test indicated that item 30 (“Shortness of breath during physical exertion?”) was problematic. It appears that this item is not easily distinguished from its own cardiovascular subscale and two other subscales (psychological fatigue and hypoglycemic subscales). This may explained by the internal consistency reliability. The Cronbach’s alpha values of the total and subscales of the K-DSC-R mostly exceeded the criterion value of 0.70; the only exception was the cardiovascular subscale, which had a Cronbach’s alpha of 0.66. In other words, the items in the cardiovascular subscale are not homogeneous. A low Cronbach’s alpha (0.69) was also found for this subscale in a psychometric study of the DSC-R [6]. The cardiovascular subscale may therefore need to be revised in future studies, including type 2 diabetes patients with varying degree of cardiovascular disease.

Known-groups validity refers to the ability of an instrument to discriminate among groups of people who are known to differ according to a hypothesized indicator [28]. As expected, the patients in the present study who were in the more-depressed groups and in the HbA1c-uncontrolled group had worse K-DSC-R scores. In particular, the effect size of the HbA1c control status on the K-DSC-R mean scores was moderate to large, implying a practical differentiation between the groups. This meaningful finding can probably be explained by a poorly controlled HbA1c being either directly or indirectly associated with micro- and macrovascular diabetic complications such as retinopathy, neuropathy, or cardiovascular problems [29]; furthermore, these complications may be perceived as symptomatic burdens to patients.

Concurrent validity refers to the correlation between a studied measure with other related measures at the same time based on a prior hypothesis with expected strength and direction [30]. A previous test of concurrent validity found that DSC-R scores were negatively correlated, with small or moderate strength, to the HRQOL subscales measured with a generic instrument [6]. The present study used a diabetes-specific HRQOL instrument (D-QOL) instead of a generic measure, so that the hypothesis of a correlation between K-DSC-R and D-QOL scores that was at moderate was supported, furthermore confirming the concurrent validity of the K-DSC-R.

A limitation of the present study was the lack of a responsiveness test to assess the ability to detect changes over time when the condition of patients is known to be altered [28]. In addition, test-retest reliability was not evaluated; the reliability assesses the extent to which an instrument yields reproducible results if it is administered repeatedly within a certain interval to a patient whose condition is stable throughout that interval [28]. Some of the acute diabetes-associated symptoms of the DSC-R, such as those associated with hyperglycemia or hypoglycemia, can fluctuate from day to day. Therefore, the most appropriate time interval for the retest should be determined before assessing the test-retest reliability of the K-DSC-R.

## Conclusions

The K-DSC-R, which comprises 29 items, exhibits acceptable factorial validity, good internal consistency reliability, item-convergent and discriminant validity, concurrent validity, and known-groups validity. It can therefore be considered reliable and valid for application in either practice or clinical trials for Korean patients with type 2 diabetes.

## Abbreviations

PRO: Patients-reported outcome; HRQOL: Health-related quality of life; DSC-R: Diabetes symptom checklist-revised; K-DSC-R: Korean version of the diabetes symptom checklist-revised; D-QOL: Diabetes-specific quality of life; CES-D: Center for epidemiologic studies-depression; HbA1c: Hemoglobin A1c; EFA: Exploratory factor analysis; CFA: Confirmatory factor analysis; CMIN/DF: Ratio of chi-square value to the degree of freedom; RMR: Root-mean-square residual; GFI: Goodness-fit index; RMSEA: Root-mean-square error of approximation; CFI: Comparative-fit index; IFI: Incremental-fit index.

## Competing interests

The authors declare that they have no competing interests.

## Author’s contribution

EHL conceptualized and deigned the study, analyzed and interpreted the data, and drafted the manuscript. KWL and RS acquired the data, interpreted the results, and revised the manuscript. FJS interpreted the results and revised the manuscript. SHM acquired and analyze the data, and helped to draft manuscript. All authors read and approved the final manuscript.

## References

[B1] International Diabetes Federation Diabetes Atlas, 5th ed http://www.idf.org/diabetesatlas/

[B2] Korean Ministry and Health Welfare Korea Health Statistics 2012: Korea National Health and Nutrition Examination Survey (KNHANES V-3) 2013 Seoul: Korean Centers for Disease Control and Prevention

[B3] American Diabetes Association Diabetes Basics http://www.diabetes.org

[B4] Posey AD Symptom perception: a concept exploration Nurs Forum 2006 41 113 124 10.1111/j.1744-6198.2006.00047.x 16879146

[B5] U.S Food and Drug Administration Guidance for Industry: Patient-Reported Outcome Measures: Use in Medical Product Development to Support Labeling Claims http://www.fda.gov/downloads/Drugs/GuidanceComplianceRegulatoryInformation/Guidances/UCM193282.pdf 10.1186/1477-7525-4-79 PMC1629006 17034633

[B6] Arbuckle RA Humphrey L Vardeva K Arondekar B Danten-Viala M Scott JA Snoek FJ Psychometric evaluation of the diabetes symptom checklist-revised (DSC-R)- a measure of symptom distress Value Health 2009 12 1168 1175 10.1111/j.1524-4733.2009.00571.x 19558371

[B7] U.S. Department of Health and Human Services FDA Center for Drug Evaluation and Research; U.S. Department of Health and Human Services FDA Center for Biologics Evaluation and Research; U.S. Department of Health and Human Services FDA Center for Devices and Radiological Health Guidance for industry: patient-reported outcome measures: use in medical product development to support labeling claims: draft guidance Health Qual Life Outcomes 2006 4 79 17034633 10.1186/1477-7525-4-79 PMC1629006

[B8] Skelly AH Leeman J Carlson J Soward ACM Burns D Conceptual model of symptom-focused diabetes care for African Americans J Nurs Scholarsh 2008 40 261 267 10.1111/j.1547-5069.2008.00236.x 18840210 PMC2567121

[B9] Schreiber JB Core reporting practices in structural equation modeling Res Social Adm Pharm 2008 4 83 97 10.1016/j.sapharm.2007.04.003 18555963

[B10] Grootenhuis PA Snoek FJ Heine RJ Bouter LM Development of a type 2 diabetes symptom checklist: a measure of symptom severity Diabet Med 1994 11 253 261 10.1111/j.1464-5491.1994.tb00268.x 8033523

[B11] Lee EH Lee YW Lee KW Kim DJ Kim SK Development and psychometric evaluation of a diabetes-specific quality of life (D-QOL) scale Diabetes Res Clin Pract 2012 95 76 84 10.1016/j.diabres.2011.08.022 21907441

[B12] Radolff LS The CES-D scale: a self report depression scale for research in the general population Appl Psychol Meas 1977 1 385 401 10.1177/014662167700100306

[B13] Cho MJ Kim KH Use of the Center for Epidemiologic Studies Depression (CES-D) scale in Korea J Nerv Ment Dis 1998 186 304 310 10.1097/00005053-199805000-00007 9612448

[B14] American Diabetes Association Standards of medical care in diabetes-2011 Diabetes Care 2011 34 Suppl 1 S11 S61 21193625 10.2337/dc11-S011 PMC3006050

[B15] Pett MA Lackey NR Sullivan JJ Making Sense of Factor Analysis: The Use of Factor Analysis for Instrument Development in Health Care Research 2003 Thousand Oaks: Sage Publications

[B16] Tabachnick BG Fidell LS Using Multivariate Statistics 2012 5 New Jersey: Pearson Education

[B17] Gable RK Instrument Development in the Affective Domain 1986 Boston: Kluwer-Nijhoff

[B18] Byrne BM Structural Equation Modeling with AMOS: Basic Concepts, Applications, and Programming 2005 Mahwah: Lawrence Erlbaum Associates Publishers

[B19] Hu LT Bentler PM Cut-off criteria for fit indexes in covariance structure analysis: conventional criteria versus new alternatives Struct Equ Modeling 1999 6 1 55 10.1080/10705519909540118

[B20] Ware JE Jr Gabdek B Methods for testing data quality, scale assumptions and reliability: The IQOLA project approach J Clin Epidemiol 1998 51 945 952 10.1016/S0895-4356(98)00085-7 9817111

[B21] Adriaanse MC Pouwer F Dekker JM Nijpels G Stehouwer CD Heine RJ Snoek FJ Diabetes-related symptom distress in association with glucose metabolism and comorbidity: the Hoorn Study Diabetes Care 2008 31 2268 2270 10.2337/dc08-1074 18728236 PMC2584176

[B22] Hu J Amoako EP Gruber KJ Rossen EK The relationships among health functioning indicators and depression in older adults with diabetes Issues Ment Health Nurs 2007 28 133 150 10.1080/01612840601096305 17365164

[B23] Matza LS Boye KS Yurgin N Validation of two generic patient-reported outcome measures in patients with type 2 diabetes Health Qual Life Outcomes 2007 5 47 10.1186/1477-7525-5-47 17672906 PMC2042494

[B24] Nunnally J Bernstein I Psychometric Theory 1994 3 New York: McGraw-Hill

[B25] Naegeli AN Stump TE Hayes RP A psychometric evaluation of the diabetes symptom checklist-revised (DSC-R) cognitive distress, fatigue, hyperglycemia, and hypoglycemia subscales in patients with type 1 and type 2 diabetes Diabetes Metab Syndr Obes 2010 3 27 30 21437073 10.2147/dmsott.s9465 PMC3047959

[B26] Klein HA Lin M-H Radford M Masuda T Choi L Lein Y Yeh Y Boff KR Cultural differences in cognition Psychol Rep 2009 105 659 674 10.2466/PR0.105.2.659-674 19928627

[B27] Lee E-O Lee S-H Validation test of Korean pain measurement tool using normal adult individuals J Korean Acad Nurs 1986 16 13 28

[B28] Fayers PM Machin D Quality of Life: The Assessment, Analysis, and Interpretation of Patient-Related Outcomes 2007 2 West Sussex: John Wiley & Sons

[B29] Kim JH Kim DJ Jang HC Choi SH Epidemiology of micro- and macrovascular complications of type 2 diabetes in Korea Diabetes Metab J 2011 25 571 577 22247898 10.4093/dmj.2011.35.6.571 PMC3253966

[B30] Terwee CB Bot SD de Boer MR van der Windt DA Knol DL Dekker J Bouter LM de Vet HC Quality criteria were proposed for measurement properties of health status questionnaires J Clin Epidemiol 2007 60 34 42 10.1016/j.jclinepi.2006.03.012 17161752

